# Intra-operative intravascular effect of the difference in colloid solutions during acute normovolemic hemodilution

**DOI:** 10.1186/s40981-021-00473-5

**Published:** 2021-09-13

**Authors:** Yoko Midorikawa, Junichi Saito, Masato Kitayama, Kentaro Toyooka, Kazuyoshi Hirota

**Affiliations:** Department of Anesthesiology, Hirosaki University Graduate School of Medicine, Zaifu-cho 5, Hirosaki, 036-8562 Japan

**Keywords:** Acute normovolemic hemodilution, Colloid, Hydroxylethyl starch, Dextran, Gynecological cancer, Blood volume

## Abstract

**Background:**

Acute normovolemic hemodilution (ANH) is used to reduce the risk of peri-operative allogeneic blood transfusion. Although crystalloid and/or colloid solutions have been used for volume replacement during ANH, no studies have examined the differences among solutions on the volume status, electrolytes, acid-base balance, and hemodynamic status during surgery with ANH.

**Methods:**

We retrospectively compared the effect of Ringer’s lactate with 3% dextran-40 (Saviosol®, DEX group) and 6% hydroxyethyl starch 130/0.4 in 0.9% sodium chloride (Voluven®, HES group) on blood hemoglobin serum electrolytes and estimated blood volume before induction of anesthesia (baseline), after ANH and after blood transfusion following surgery in patients undergoing open gynecological surgery (*n* = 111 and 67, respectively). The primary outcomes were the changes in hemoglobin and electrolytes after ANH.

**Results:**

There were no differences in hemoglobin or electrolytes between the two groups at baseline. Postoperative hemoglobin was significantly higher (11.0 ± 1.5 g/dL vs 9.9 ± 1.3 g/dL) (mean ± SD) in the DEX group than in the HES group (*p* = 0.03). Postoperative potassium was significantly decreased from the baseline both in the DEX group (137.9 ± 2.5 mmol/L vs 136.3 ± 2.7 mmol/L) and in the HES group (138.3 ± 2.0 mmol/L vs 137.8 ± 2.5 mmol/L) (*p* < 0.001 for both); however, it was significantly higher than in the DEX group after surgery (*p* < 0.001). Estimated blood volume after surgery was significantly increased after ANH in both groups; however, it was larger in the HES group than in the DEX group.

**Conclusions:**

Postoperative hemoglobin and potassium were significantly higher, and estimated blood volume was significantly smaller in the DEX than in the HES group.

## Introduction

Acute normovolemic hemodilution (ANH) is one of the autologous blood transfusion techniques that are used to reduce the risk of peri-operative allogeneic blood transfusion in both cardiac surgery [1, 2] and non-cardiac surgery [3–5]. The coronavirus disease 2019 (COVID-19) pandemic has had a negative impact on blood donations, resulting in an allogeneic blood supply shortage and cancelations of surgery worldwide [6, 7]. In this situation, the use of ANH can be a key strategy to reduce the demand for allogeneic transfusions [8].

A rapid infusion of a crystalloid and/or colloid solution is necessary to maintain euvolemia during acute hemodilution. Compared to crystalloid solutions, colloid solutions, i.e., those containing albumin, dextran, gelatin, and hydroxyethyl starch, provide some advantages for maintaining a patient’s hemodynamic state during acute blood loss [9, 10]. In addition to the differences in types of colloid, the composition of the fluids differs among the colloid solutions, depending on whether the solution is Ringer’s-based or saline-based.

Ringer’s lactate with 3% dextran-40 (Saviosol®; Otsuka Pharmaceutical Factory, Naruto, Japan) had been used until 2014; then, it was replaced with 6% hydroxyethyl starch 130/0.4 in 0.9% sodium chloride (Voluven®; Otsuka Pharmaceutical Factory). To the best of our knowledge, there has been no investigation on the impacts of colloid solutions in patients receiving ANH. We compared the effect of these two colloid solutions on the volume status, electrolytes, acid-base balance, and hemodynamic status during surgery. We conducted the present retrospective study to assess the impact of colloid solutions on the volume status, electrolytes, acid-base balance, and hemodynamic status during ANH.

## Methods

The study adheres to the STROBE statement. The retrospective study design and protocol were approved by the Ethics Committee of Hirosaki University Graduate School of Medicine and publicized on our hospital homepage (2021-039). As the study was retrospective, the requirement of patients’ written informed consent was waived by the Ethics Committee. The patient characteristics and peri-operative data were collected from anesthetic and medical records of patients with gynecological cancer (≥ 20 years old) who received ANH during open abdominal surgery at Hirosaki University Hospital during the period from April 1, 2013, to March 31, 2016. Patients who did not receive ANH or were infused with both Saviosol® and Voluven® during surgery were excluded from the study. We divided the patients into two groups based on the colloid solutions: the Saviosol® (DEX) group and the Voluven® (HES) group.

### Data collection

We collected the following data from the patients’ medical records: age, height, body weight, body mass index (BMI), and the use of peri-operative allogeneic blood transfusion (red blood cells, fresh frozen plasma, and platelet concentration until postoperative day [POD] 3). The following data were collected from the patients’ anesthesia records: the American Society of Anesthesiologists-Physical Status (ASA-PS); the amounts of ephedrine and/or phenylephrine administered; the amount of ANH blood; the intra-operative total fluid volume of crystalloid and colloid, i.e., Voluven® and Saviosol®; the intra-operative urine out; the intra-operative estimated blood loss; and the durations of surgery and anesthesia. Peri-operative laboratory values including the hemoglobin concentration (Hb), platelet count, serum sodium (Na), potassium (K), chloride (Cl), and base excess (BE) were obtained at three time points: before the induction of anesthesia, after acute hemodilution, and after the surgery (after a re-transfusion of ANH blood) in the post-anesthesia care unit.

We also collected the data of the patients’ peri-operative kidney function (serum creatinine [sCre] and the estimated glomerular filtration rate [eGFR]) within 14 days before surgery and on POD-1 from the electronic medical records. The primary outcomes were the changes in Hb and changes in electrolytes during ANH.

### Calculation of blood volume and changes in blood volume

Blood volume (BV) was calculated using the formula [11]:

$$ \mathrm{BV}\ \left(\mathrm{dL}\right)=\left(0.0003561\times {\mathrm{height}}^3\ \left(\mathrm{cm}\right)+\left[33.08\times \mathrm{body}\ \mathrm{weight}\ \left(\mathrm{kg}\right)+183\right]\right)/100 $$

The patient’s BV after surgery was calculated by first estimating the total Hb mass in the circulation at baseline “Hbmass(0)” as being equal to the product of BV(0) and the Hb concentration at baseline, “Hb(0).” Losses from Hb mass were then subtracted for measurement, and the BV(1) was obtained by dividing this difference by a freshly taken Hb value after surgery [12, 13].

$$ \mathrm{Hb}\mathrm{mass}(0)=\mathrm{BV}(0)\times \mathrm{Hb}(0) $$

$$ \mathrm{Hb}\mathrm{mass}\left(\mathrm{loss}\right)=\mathrm{blood}\ \mathrm{loss}\times \mathrm{Hb}(1) $$

$$ \mathrm{BV}(1)=\left[\mathrm{Hbmass}(0)-\mathrm{Hbmass}\left(\mathrm{loss}\right)\right]/\mathrm{Hb}(1) $$

$$ \mathrm{Changes}\ \mathrm{in}\ \mathrm{BV}=\mathrm{BV}(1)-\mathrm{BV}(0) $$

### General anesthesia and ANH

All open abdominal gynecological surgeries were managed under total intravenous anesthesia: propofol, ketamine, remifentanil and/or fentanyl, and rocuronium. ANH was conducted in patients who were at risk of a blood loss of ≥ 500 mL during surgery, at the request of the gynecological surgeon.

Blood withdrawal from the patients was started just after induction of anesthesia and before skin incision. The whole blood from a central venous line was collected into standard blood collection bags containing a citrate phosphate dextrose solution. To maintain the patient’s euvolemia and mean arterial pressure ≥ 60 mmHg, the withdrawn whole blood was replaced by the same volume of colloid solution (Voluven® or Saviosol®). To maintain mean arterial pressure ≥ 60 mmHg, ephedrine and/or phenylephrine were administered during blood withdrawal. The withdrawn blood volume for ANH was targeted at 400–800 mL and decided by the attending anesthesiologist and supervisor of the operating theater to prevent Hb < 8.0 g/dL after acute hemodilution. ANH procedure took about 20 to 25 min to collect 800 mL of blood. ANH blood bags were shaken 60–80 rpm in the room temperature. Subsequently, all patients received acetate Ringer’s solution and/or colloid solution during anesthesia. When a specimen was removed or surgical hemostasis was performed, all of the collected ANH blood was reinfused to the patient in the operating theater.

### Statistical analyses

We used the D’Agostino and Pearson normality test to determine whether all variables showed a Gaussian distribution. Continuous data are presented as the mean ± standard deviation (SD) or the median [interquartile range, IQR]. Categorical data are presented as the number (%). Student’s *t*-test or the Mann-Whitney *U*-test was used for analyzing continuous variables, and the Chi-squared test or Fisher’s exact test was used for analyzing categorical variables. We conducted a repeated two-way analysis of variance (ANOVA) with Tukey’s multiple comparisons tests to evaluate the changes in all variables in the peri-operative period. A repeated two-way ANOVA with Sidak’s multiple comparisons test was conducted to assess the difference in each time point between the two groups. Probability (*p*)-values < 0.05 were considered significant. All reported *p*-values are two-tailed. Statistical analyses were performed using GraphPad Prism 7.02 (GraphPad Software, San Diego, CA, USA).

No prior sample size calculation was performed in this study. However, our sample size (111 vs 67) had 99.1% power to detect 300 mL absolute difference in BV after surgery between the DEX and the HES group at the 0.05 level. Sample size calculation was performed using EZR software ver. 1.37 (Saitama Medical Center, Jichi Medical University, Saitama, Japan).

## Results

Of the 189 patients initially included, 11 were excluded from the analysis because both Saviosol® and Voluven® were infused during surgery. Subsequently, data from 178 patients (DEX group, *n* = 111 and HES group, *n* = 67) were analyzed. There were no differences in the patients’ profiles, volume of ANH, estimated blood loss, allogeneic transfusion, and duration of surgery (Table 1).

**Table 1:** The patients’ characteristics and intra-operative data

	HES, ***n*** = 67	Dex, ***n*** = 111	***p***-value
Age, years	56 (12)	54 (13)	0.443
Height, cm	154.8 (4.7)	155.0 (6.0)	0.598
Body weight, kg	57.3 (11.9)	60.0 (13.6)	0.182
BMI	24.0 (5.1)	25.0 (5.6)	0.259
ASA-PS			
1	4 (6.0)	7 (6.3)	0.899
2	56 (83.6)	95 (85.6)	
3	7 (10.4)	9 (8.1)	
Diagnosis			
Ovarian cancer, *n* (%)	11 (16.4)	17 (15.3)	0.724
Uterine body cancer, *n* (%)	40 (59.7)	61 (55.0)	
Cervical cancer, *n* (%)	12 (17.9)	27 (24.3)	
Others, *n* (%)	4 (6.0)	6 (5.4)	
Amount of ANH			
400–599 mL	6 (9.0)	8 (7.2)	0.654
600–799 mL	5 (7.5)	6 (5.4)	
≥ 800 mL	56 (83.6)	97 (87.4)	
Anesthetics			
PRK	11 (16.4)	17 (15.3)	0.835
PFK	56 (83.6)	94 (84.7)	
Total fluid infusion	2400 (2000, 2700)	2700 (2300, 3250)	< 0.001
Crystalloid	1500 (1000, 1813)	1700 (1350, 2350)	< 0.001
Colloid	1000 (1000, 1000)	1000 (1000, 1000)	0.718
RBC	2 (3.0)	1 (0.9)	0.557
FFP	0 (0)	0 (0)	1.000
PC	0 (0)	0 (0)	1.000
Estimated blood loss, g	430 (260, 705)	445 (200, 655)	0.724
Urine out, mL	185 (129, 300)	223 (133, 350)	0.278
Duration of surgery, min	191 (53)	200 (56)	0.310
Duration of anesthesia, min	253 (58)	266 (61)	0.149

The data are mean (SD) or median (25%, 75%). *ANH* acute normovolemic hemodilution; *ASA-PS* American Society of Anesthesiologists-Physical Status; *BMI* body mass index; *Dex* dextran; *FFP* fresh frozen plasma; *HES* hydroxylethyl starch; *PC* platelet concentrates; *PFK* propofol, fentanyl, ketamine; *PRK* propofol, remifentanil, ketamine; *RBC* red blood cells

### Volume of infusion, hemoglobin concentration, and estimated blood volume

**Fig. 1:**
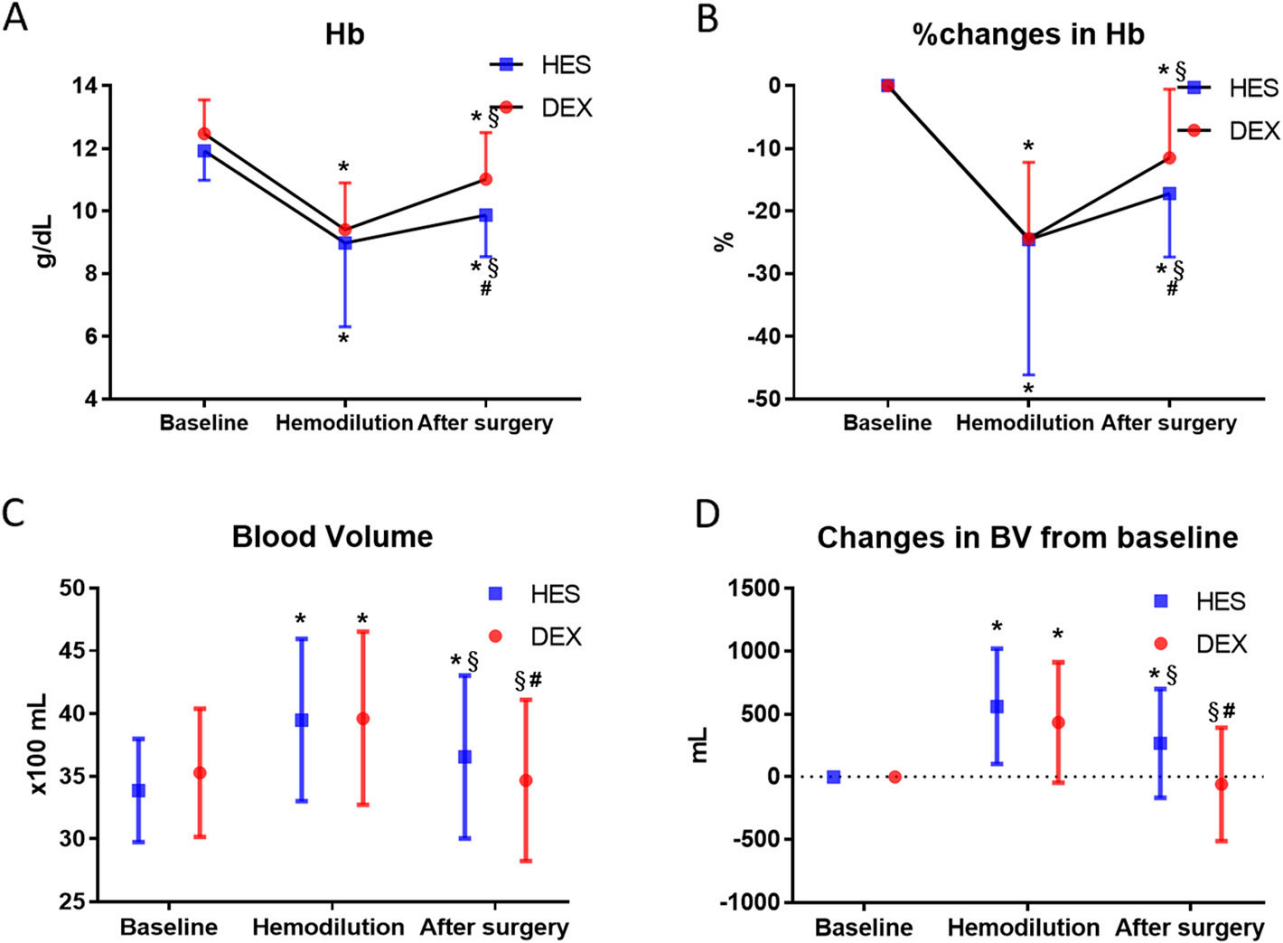
Difference in hemoglobin concentration and blood volume between the HES group and the DEX group. **A** Changes in hemoglobin concentration. **B** %Changes in hemoglobin concentration. **C** Changes in blood volume. **D** Changes in blood volume from the baseline value; HES, hydroxylethyl starch; DEX, dextran; Hb, hemoglobin concentration; BV, blood volume; *p < 0.01 vs. baseline. ^§^*p* < 0.01 vs. hemodilution. ^#^*p* < 0.01 vs. DEX

The volume of colloid solution was comparable between the two groups. On the other hand, both the volume of crystalloid and the total volume of colloid and crystalloid solution were significantly larger in the DEX than in the HES group (*p* < 0.001 for both, Table 1). Hemoglobin concentration significantly decreased after ANH from the baseline, which was increased at the end of surgery in both groups. However, it was significantly higher in the DEX group than in the HES group after surgery (*p* = 0.03, Table 2). Estimated blood volume was significantly increased after ANH compared with baseline (*p* < 0.01) and decreased after surgery in both groups (Fig. 1c). The BV after surgery in the DEX group was comparable to the baseline, whereas it was significantly larger than baseline in the HES group (*p* < 0.01), and significantly larger than in the DEX group (*p* < 0.01). Platelet count was significantly decreased after ANH and remained unchanged in both groups, and there were no differences between the two groups during the study period.

**Table 2:** Changes in variables during surgery

		Baseline	After ANH	After surgery	*F*	*p*-value
Hb, g/dL	DEX	12.5 ± 1.1	9.4 ± 1.5*	11.0 ± 1.5*^§#^	3.520	0.0307
	HES	11.9 ± 0.9	9.0 ± 2.7*	9.9 ± 1.3*^§^		
Plt, 10^4^/μL	DEX	24.5 ± 6.6	19.7 ± 6.0*	19.9 ± 6.1*	0.526	0.5913
	HES	22.6 ± 6.4	18.4 ± 5.2*	18.3 ± 5.0*		
Na, mmol/L	DEX	137.9 ± 2.5	137.0 ± 2.2*^#^	136.3 ± 2.7*^§#^	7.962	0.0004
	HES	138.3 ± 2.0	138.7 ± 2.2	137.8 ± 2.5^§^		
K, mmol/L	DEX	3.7 ± 0.2	3.5 ± 0.3*	3.7 ± 0.4^§^	1.244	0.2896
	HES	3.7 ± 0.2	3.4 ± 0.2*	3.6 ± 0.3^§^		
Cl, mmol/L	DEX	107.6 ± 2.3	109.8 ± 2.3*^#^	109.0 ± 3.0*^§#^	22.56	< 0.0001
	HES	107.7 ± 2.0	112.3 ± 2.1*	111.0 ± 2.4*^§^		
BE, mg/dL	DEX	1.8 ± 1.7	0.6 ± 1.6*^#^	0.2 ± 1.7*^#^	7.100	0.0010
	HES	0.8 ± 1.8	− 1.2 ± 1.7*	− 1.3 ± 1.6*		
Lac, mmol/L	DEX	0.8 ± 0.2	1.2 ± 0.5*^#^	1.0 ± 0.5*^§#^	23.03	< 0.0001
	HES	0.7 ± 0.2	0.6 ± 0.2*^#^	0.8 ± 0.5^§#^		

The data are mean ± SD

*p < 0.01 vs. baseline, ^§^*p* < 0.01 vs. after ANH, ^#^*p* < 0.01 vs. HES

*DEX* dextran, *HES* hydroxyethyl starch, *BE* base excess, *Cl* chloride, *Hb* hemoglobin, *K* potassium, *Lac* lactate, *Na* sodium, *Plt* platelet count

### Electrolytes

Serum Na significantly decreased after ANH and after surgery compared with the baseline in the DEX group. On the other hand, serum Na unchanged after ANH, and decreased after surgery compared before ANH in the HES group. Serum Na in the DEX group was significantly lower than the HES group after ANH and surgery (*p* < 0.001 for both, Table 2). Serum K was significantly decreased after ANH and after surgery compared with baseline in both groups (*p* < 0.001 for both, Table 2), and there were no differences between the two groups during the study period. Serum chloride was significantly increased in both group after ANH and after surgery compared with baseline in both groups (*p* < 0.01 for both), but it was significantly higher in the HES group than in the DEX group (*p* < 0.01, Table 2).

### Base excess and lactate

BE was significantly higher in the DEX group compared with the HES group at baseline (*p* < 0.001, Table 2). BE significantly decreased after ANH and after surgery. It was significantly higher in the DEX group compared with the HES group (*p* < 0.001). Serum lactate was similar between the groups at baseline, which was significantly increased after ANH and decreased after surgery in the DEX group; inversely, it was significantly decreased after ANH and increased after surgery in the HES group. Serum lactate was significantly higher in the DEX group than in the HES group after ANH and after surgery (*p* < 0.001 for both).

### Vasoactive drugs

The rate and amounts of vasoactive drugs (ephedrine and phenylephrine) used during surgery were comparable between the two groups.

### Peri-operative kidney function

As summarized in Table 3, the mean pre-operative sCre and eGFR values were comparable between the two groups (*p* = 0.866). On POD-1, the mean sCre values were significantly decreased (*p* < 0.001) and the mean eGFR values were significantly increased (*p* < 0.001). These values were comparable between the two groups (*p* = 0.636).

**Table 3:** Changes in renal function from before surgery to POD-1

		Pre-operative	POD-1	***p***-value
sCre, mg/dL	Saviosol	0.58 [0.53, 0.65]	0.52 [0.47, 0.58]	< 0.001
	Voluven	0.58 [0.53, 0.62]	0.50 [0.47, 0.56]	< 0.001
eGFR, mL/kg/1.73 m^2^	Saviosol	84.8 [73.3, 95.3]	94.3 [79.5, 107]	< 0.001
	Voluven	81.1 [75.6, 94.3]	95.9 [82.8, 109.4]	< 0.001

*eGFR* estimated glomerular filtration rate, *POD* postoperative day, *sCre* serum creatinine

## Discussion

The results of our retrospective analyses revealed that the difference in colloid solutions during ANH had significant impacts on the patients’ volume status, electrolytes, and acid-base balance during surgery.

Hemoglobin levels after surgery in the DEX group was significantly higher than in the HES group, despite lager volume of fluids administered in the DEX group than in the HES group. This would result from the different effect between DEX and HES on plasma volume. HES remained in the blood vessels longer than DEX because of its longer half-life, which was supported by increased blood volume in the HES group after surgery. The product monographs of Saviosol® and Voluven® hold that the expansion effect lasts for 2–3 and 4–6 h, respectively. The turnover of HES based on weight/volume measurements identified the elimination half-life (T1/2) of 12.1 h [14], which is longer than that of Dextran 40 (elimination T1/2 10 h) [15]. Another study revealed that when Ringer’s acetate was infused after HES, the elimination half-life of acetate Ringer’s solution was five times longer compared to when acetate Ringer’s solution was infused alone (497 min vs. 88 min, *p* < 0.001) [16]. In addition to the direct expansion effect of HES, an interaction with Ringer’s solution might have contributed to our present results.

In both the present DEX and HES groups, the BV after hemodilution increased; the increases were similar between the two groups. The increases in BV in the two groups were greater than expected. The explanation of this phenomenon is that a decrease in blood pressure during the induction of anesthesia increased the plasma volume [17, 18]. The calculated increase in plasma volume without fluid infusion during the induction of anesthesia was 10–15% [17], corresponding to a 310 to 460 mL increase in plasma volume. According to the Starling mechanism [19], transcapillary fluid exchange depends on a balance between hydrostatic and oncotic pressure gradients. A reduction of blood pressure induces an acute reduction of the capillary hydrostatic pressure, accompanied by an increase in plasma volume.

Our analyses also revealed that acute hemodilution with 1000 mL of Voluven® increased the patients’ Cl by nearly 5 mmol/L and decreased their BE by 2 mmol/L, but on POD-1 these changes had not worsened the patients’ kidney function. A retrospective study supports our findings; ANH conducted with Voluven® did not increase the incidence of acute kidney injury after major abdominal surgery (4.8% vs. 8.0%, *p* = 0.20), even though ANH induced transient hyper-chloridemia acidosis during surgery [20]. In addition, a randomized controlled trial revealed that balanced 6% HES 130/0.4 (Tetraspan®) and Voluven® showed equal effects for the hemodynamic stabilization of patients undergoing major urologic surgery, without any significant impact on renal function until POD-3 [21]. Additionally, a recent large retrospective study from Japan [22] revealed that the incidence of acute kidney injury was 6.2% (548/8823) in patients receiving Voluven® and 5.6% (492/8823) in controls (OR 1.12; 95% CI 0.99–1.27; *p* = 0.07) and Voluven® administration was not associated with worsening acute kidney injury stage (OR 0.89; 95% CI 0.79–1.01; *p* = 0.08). Interestingly, the incidence of renal replacement therapy was lower in patients receiving Voluven® than that in controls (0.2% vs 0.4%, respectively; OR 0.51; 95% CI 0.29–0.91; *p* = 0.02). These results suggest that transient hyper-chloridemia acidosis is tolerable if volume expansion using colloid solution is well indicated.

A possible mechanism of renal injury-associated hyper-chloridemia is that hyper-chloridemia induces vasoconstriction in afferent arterioles in the kidneys and decreases in renal blood flow and perfusion, thereby causing reductions of the glomerular filtration rate and urine output [23]. Additionally, acidosis may increase the afferent arteriolar resistance in the kidneys. Hyper-chloridemia acidosis thus has a potential to reduce renal function. As the clinical impact of infusion-related hyper-chloridemia acidosis has not been fully elucidated, further randomized controlled studies with large sample sizes are required to determine the relationships between infusion-related hyper-chloridemia acidosis and peri-operative renal function and other clinical outcomes of surgical patients.

Our study has some limitations to address. The setting of ANH was not fixed due to a retrospective design. The different setting of ANH could have some impacts on the glycocalyx degeneration and volume status after ANH and, thus, would lead to a bias of the results. In addition, we did not evaluate long-term outcomes including renal function and the survival rate. Further studies with greater numbers of patients are necessary to determine the safety of colloid solutions during ANH in higher-risk patients undergoing major abdominal surgery.

## Conclusions

This retrospective study suggested that the difference in colloid solution during ANH had significant impacts on the volume status, electrolytes, and acid-base balance during surgery for gynecological cancer.

## Data Availability

The datasets used and/or analyzed during the current study are available from the corresponding author on reasonable request.
